# Increasing *in situ* bioremediation effectiveness through field-scale application of molecular biological tools

**DOI:** 10.3389/fmicb.2022.1005871

**Published:** 2023-02-10

**Authors:** Andrew S. Madison, Skyler J. Sorsby, Yingnan Wang, Trent A. Key

**Affiliations:** ^1^Golder Associates USA Inc., (Currently WSP USA Inc.), Marlton, NJ, United States; ^2^Imperial Oil Limited, Calgary, AB, Canada; ^3^ExxonMobil Environmental and Property Solutions Company, Spring, TX, United States

**Keywords:** bioremediation, molecular biological tools, contamination, groundwater contaminant characterization, bioremediation framework, contaminated site assessment, contaminated site cleanup, biodegradation

## Abstract

Leveraging the capabilities of microorganisms to reduce (degrade or transform) concentrations of pollutants in soil and groundwater can be a cost-effective, natural remedial approach to manage contaminated sites. Traditional design and implementation of bioremediation strategies consist of lab-scale biodegradation studies or collection of field-scale geochemical data to infer associated biological processes. While both lab-scale biodegradation studies and field-scale geochemical data are useful for remedial decision-making, additional insights can be gained through the application of Molecular Biological Tools (MBTs) to directly measure contaminant-degrading microorganisms and associated bioremediation processes. Field-scale application of a standardized framework pairing MBTs with traditional contaminant and geochemical analyses was successfully performed at two contaminated sites. At a site with trichloroethene (TCE) impacted groundwater, framework application informed design of an enhanced bioremediation approach. Baseline abundances of 16S rRNA genes for a genus of obligate organohalide-respiring bacteria (i.e., *Dehalococcoides*) were measured at low abundances (10^1^–10^2^ cells/mL) within the TCE source and plume areas. In combination with geochemical analyses, these data suggested that intrinsic biodegradation (i.e., reductive dechlorination) may be occurring, but activities were limited by electron donor availability. The framework was utilized to support development of a full-scale enhanced bioremediation design (i.e., electron donor addition) and to monitor remedial performance. Additionally, the framework was applied at a second site with residual petroleum hydrocarbon (PHC) impacted soils and groundwater. MBTs, specifically qPCR and 16S gene amplicon rRNA sequencing, were used to characterize intrinsic bioremediation mechanisms. Functional genes associated with anaerobic biodegradation of diesel components (e.g., naphthyl-2-methyl-succinate synthase, naphthalene carboxylase, alkylsuccinate synthase, and benzoyl coenzyme A reductase) were measured to be 2–3 orders of magnitude greater than unimpacted, background samples. Intrinsic bioremediation mechanisms were determined to be sufficient to achieve groundwater remediation objectives. Nonetheless, the framework was further utilized to assess that an enhanced bioremediation could be a successful remedial alternative or complement to source area treatment. While bioremediation of chlorinated solvents, PHCs, and other contaminants has been demonstrated to successfully reduce environmental risk and reach site goals, the application of field-scale MBT data in combination with contaminant and geochemical data analyses to design, implement, and monitor a site-specific bioremediation approach can result in more consistent remedy effectiveness.

## 1. Introduction

Natural or enhanced bioremediation is increasingly utilized in contaminated site management strategies. Bioremediation methods can be favorable strategies for contaminated site management as natural microorganisms present at impacted sites are capable of reducing (degrading or transforming) concentrations of a wide range of pollutants in soil and groundwater (Bouwer and Zehnder, 1993; Wiedemeier et al., 1999; Bombach et al., 2010). Traditional design and implementation of bioremediation strategies, including enhanced bioremediation treatment technologies or monitored natural attenuation (MNA) strategies, rely on contaminant concentrations and spatiotemporal trends, field-scale geochemical data, and/or lab-scale biodegradation studies. While useful for remedial decision-making, these evaluations are largely indirect indicators of *in situ* microbial activity and bioremediation potential (Wiedemeier et al., 1999; Rittmann and McCarty, 2001; Key et al., 2014; Lawson et al., 2019). Additional insights can be gained through the field-scale application of Molecular Biological Tools (MBTs) to analyze field samples and directly measure biomarkers (e.g., genes) associated with contaminant-degrading or transforming microorganisms and associated enzymes, which can vary spatially and temporally (Interstate Technology and Regulatory Council, 2013; Taggart and Clark, 2021).

Over the past 20 years, MBTs have been increasingly utilized, due to scientific advancement and decreased analytical cost, to directly assess microbiological processes at contaminated sites (Wilson et al., 1999; Madsen, 2000; Beller et al., 2002; Winderl et al., 2007; Cupples, 2008; Gedalanga et al., 2016; Wilson et al., 2019; Taggart and Clark, 2021; Wetterstrand, 2022). MBTs, such as quantitative polymerase chain reaction (qPCR) and 16S rRNA gene amplicon sequencing [often termed within the contaminated site management community as Next Generation Sequencing (NGS)], can be used to provide actionable data to inform site management. Many publications, including those in this research topic issue, present the advantages of employing MBTs in concert with contaminant chemistry and geochemistry evaluations to reduce site uncertainties and better characterize subsurface microbiology (Rittmann and McCarty, 2001; Amos et al., 2008; Busch-Harris et al., 2008; Bombach et al., 2010; Zhang et al., 2016; Bouchard et al., 2018; Lawson et al., 2019). Nonetheless, application, implementation strategies, and data interpretation of MBTs can be inconsistent in practice, which could potentially become barriers to increased uptake and acceptance within the contaminated site management community.

A framework was developed to demonstrate how MBTs could be consistently applied at the field-scale to reduce uncertainty of subsurface biological processes and support decision-making using site-specific biogeochemical data (Key et al., 2022). This framework was successfully performed at two contaminated sites to assess bioremediation capacity and guide remedial decision-making. The framework was applied to a site with trichloroethene (TCE) impacted groundwater, enumeration of 16S rRNA genes for a genus of obligate organohalide-respiring bacteria (i.e., *Dehalococcoides*) along with contaminant trends and geochemical data were utilized to develop a full-scale enhanced bioremediation design and to monitor remedial performance. At a second site with residual petroleum hydrocarbon (PHC) impacted soils and groundwater, MBTs, specifically qPCR and 16S rRNA gene amplicon sequencing (NGS), were used to identify and characterize contaminant-degrading microorganisms and associated biodegradation activities to assess the feasibility of a MNA approach within the downgradient plume and an enhanced bioremediation approach (i.e., biostimulation) within the source area.

## 2. Materials and methods

### 2.1. Field-scale framework

The field-scale framework structure applied to the sites is detailed in Key et al. (2022) and consists of two elements: (1) following a staged process and (2) using a multiple lines of evidence (MLOE) approach for data generation and interpretation to establish and address site-specific bioremediation objectives (SSBOs). The objective of the framework is to define a systematic approach to assess applicability of bioremediation, support Conceptual Site Model (CSM) development by measuring *in situ* biogeochemical processes, design a bioremediation approach, and measure bioremediation performance using field-scale MBT data. Many of the details applied through the framework at these two sites, such as tool selection and sampling plan development, were aligned with ASTM E3354 Standard Guide for Application of Molecular Biological Tools to Assess Biological Processes at Contaminated Sites (ASTM International, 2022). It should be noted that while this framework was found to be both pragmatic and effective to support site decision-making at the two sites described here, other frameworks or approaches may be applicable.

The framework staged process begins with the **Framework Commencement** where historical site data is reviewed to understand the current site setting, nature and extent of contamination, and site-specific risk drivers and establish SSBOs. Evaluation of existing chemical data and hydrogeological data should be integrated to a coherent CSM summarizing contaminant fate and transport processes, and receptor exposure to inform the rationale for and scope of generating MBT data to support bioremediation. The SSBOs should be defined to focus on the intent of MBT data generation, such as determining whether the generated data will be used to support a natural or enhanced bioremediation strategy and develop biodegradation data objectives. In **Stage 1: Assessment**, the objective is to identify potential biodegradation or biotransformation processes by performing targeted sampling for MLOE (contaminants, geochemical indicator parameters, and MBTs) at locations across the area of interest and across intrinsic contaminant concentration or geochemical gradients. Spatial and temporal contaminant trends provide empirical evidence of attenuation and biodegradation, while geochemical results help to elucidate potential associated biodegradation mechanisms and potential electron acceptor/donor limitations to intrinsic biodegradation. MBTs are evaluated to determine the presence and abundance of contaminant-degrading microorganisms or enzymes, and the inferred extent of intrinsic biodegradation. When the results of the assessment stage suggest a bioremediation approach is feasible, the contaminant, geochemical, and MBTs results are evaluated during the **Stage 2: Design** to support determination and design a site-specific bioremediation strategy. For natural bioremediation strategies, these data should provide conclusive evidence to support occurrence of intrinsic bioremediation across the area of interest. Additionally, the contaminant, geochemical, and MBT data should guide the design of the natural bioremediation monitoring program. If the results of the Assessment Stage indicate enhanced bioremediation (i.e., biostimulation) is warranted to meet site goals, the contaminant, geochemical, and MBTs data can guide approaches to enhance attenuation by adding a site-specific amendment [e.g., electron donor (emulsified vegetable oil [EVO], lactate, formate), electron acceptor (oxygen, nitrate, and sulfate), nutrients (nitrogen, phosphorous, trace metals, and vitamins)], and/or microbial cultures (e.g., bioaugmentation) to areas of residual impacts. Following implementation of the natural or enhanced bioremediation strategy, the **Stage 3: Performance Monitoring** includes generating and monitoring contaminant, geochemical, and MBT data to assess remedy progress toward objectives. Because spatiotemporal contaminant trends can be confounded by complex fate and transport processes that complicate biodegradation and remedy performance determinations, MBTs and geochemical data can be helpful to demonstrate biogeochemical effects of the remedy, and in some cases serve as a leading indicator of remedy performance.

### 2.2. Site 1 description

**Figure 1:**
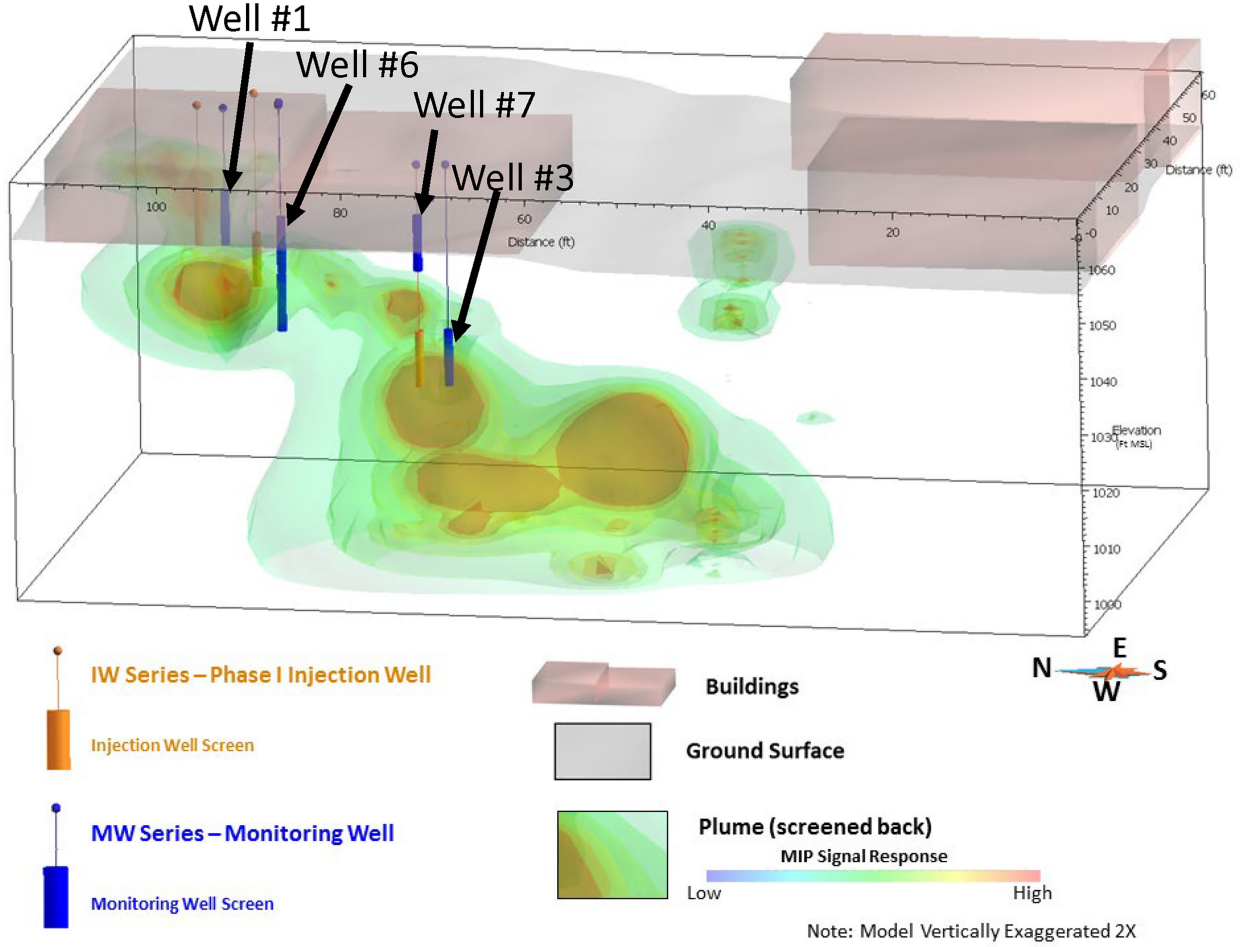
Site 1 TCE plume configuration based on MIP results. Pilot study injection wells are shown in orange and pilot study performance monitoring wells are shown in blue.

The framework approach was applied and validated at a former manufacturing site where trichloroethene (TCE) was used during manufacturing activities that resulted in impacted soil and groundwater. Groundwater impacts extend across three general hydrostratigraphic depth zones (termed shallow, intermediate, and deep) within the underlying eolian loess deposits. Hard-pan horizons characterized by comparatively lower hydraulic conductivity separate the hydrostratigraphic zones. Within each zone, groundwater generally flows to the south. Site investigation activities, primarily high-resolution site characterization using membrane interface probe (MIP) technology, identified the transport of TCE-impacted groundwater is controlled by lateral migration along the hard-pan layers until isolated areas of vertical fractures are encountered that facilitate downward transport into the underlying hydrostratigraphic zone. These transport mechanisms result in a stair-shaped plume, as shown by MIP results in Figure 1.

### 2.3. Site 2 description

**Figure 2:**
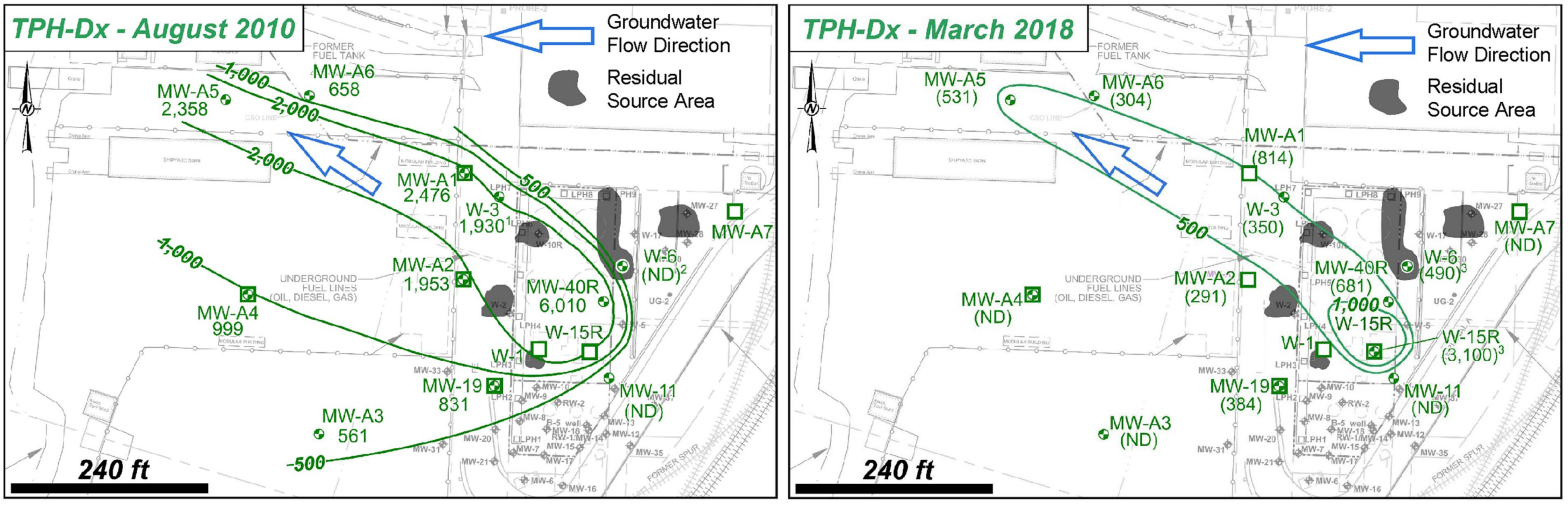
Site 2 overview showing shallow groundwater TPH-Dx plume contraction between August 2010 and March 2018.

The field-scale framework was applied to a former PHC bulk storage terminal that operated from at least the 1920s to the 1990s. Soil and groundwater impacts have been investigated across the site and have observed free-phase, light non-aqueous phase liquid (LNAPL) in shallow soil and in the shallow water table zone which generally fluctuates one to five feet below the ground surface. Multiple source areas characterized by observations of LNAPL are present within the former site footprint (Figure 2). Shallow impacts are vertically contained within the anthropogenic fill unit that underlies the site to depth of more than 20 feet below the ground surface. The anthropogenic fill unit comprises a heterogeneous mixture of silts, sands, wood fragments, and brick debris deposits with hydraulic conductivity varying from 4 to 84 feet per day (ft/day). Shallow, unconfined groundwater generally flows to the northwest.

The primary contaminants at the site are PHC, primarily diesel range organics (TPH-Dx) and gasoline range organics (TPH-Gx). TPH-Dx is detected most consistently above regulatory thresholds across the site in soil and groundwater and beyond the site within the shallow, dissolved-phase groundwater plume (Figure 2). TPH-Gx is detected only in soils and groundwater within the residual source areas. When detected in soil and groundwater, aromatic hydrocarbons such as benzene, toluene, ethylbenzene, and total xylenes (BTEX) are near detection limits and below cleanup criteria. Excavation of impacted source soils occurred from the 1980s to the 2000s. Free-product removal at recovery wells and installation and operation of LPH-recovery and soil-vapor extraction trenches also were performed and reached technological mass removal limits. However, impacts above regulatory standards are still present in soil and groundwater.

### 2.4. Chemical sample collection and analysis

Groundwater sampling was performed at Sites 1 and 2 during the field-scale framework stages in a systematic approach to characterize contaminant and geochemical conditions within impacted areas, including source and downgradient plume areas, and from background sample locations for comparative purposes. Groundwater samples were collected using low-flow groundwater sampling methods (Puls and Barcelona, 1996).

Groundwater sampling at Site 1 was performed to establish contaminant concentrations and geochemical conditions across the lateral and vertical extent of the groundwater plume at groundwater monitoring wells during the Assessment Stage baseline characterization (nine monitoring wells), Design Stage pilot-scale implementation (four monitoring wells), and remedy Performance Monitoring Stage (nine monitoring wells). Contaminant analyses included volatile organic compounds [VOCs; U.S. Environmental Protection Agency (EPA) Method 8260] and ethene (US EPA Method RSK-175), a non-toxic reductive dechlorination end product. Geochemical analyses included general biogeochemical indicators [pH, specific conductivity, temperature, dissolved oxygen, oxidation–reduction potential (ORP), and alkalinity (SM 2320B)], potential bacterial electron acceptors (nitrate and sulfate by US EPA Methods 353.3 and 300.0 respectively), potential by-products of anaerobic microbial respiration [ferrous iron (SM21 3500FE B), sulfide (SM 4500 S2 F), and methane (US EPA Method RSK-175)], and organic carbon growth substrate availability [i.e., total organic carbon (TOC) by SM 5310B]. Samples for contaminant and biogeochemical indicators were analyzed by a certified, commercial analytical laboratory (Pace Analytical Services, LLC).

Groundwater samples were collected at Site 2 to supplement historical contaminant and biogeochemical indicator data from the background, source area, and downgradient plume monitoring wells during the Assessment Stage baseline characterization (seven monitoring wells) and from six pilot study monitoring wells during the Performance Monitoring Stage. Contaminant analyses included PHCs (TPH-Dx and TPH-Gx by NWTPH) and BTEX (US EPA Method 8260C). Biogeochemical indicator analyses included general biogeochemical indicators [pH, specific conductivity, temperature, dissolved oxygen, ORP, and alkalinity (SM 2320B)], potential bacterial electron acceptors (nitrate and sulfate by US EPA Method 300.0), and potential by-products of anaerobic microbial respiration [ammonia (SM 4500 NH3-C), iron and manganese (US EPA Method 6020), and methane (US EPA Method RSK-175)]. Samples for contaminant and biogeochemical indicators were analyzed by a certified, commercial analytical laboratory (Eurofins Calscience, LLC).

### 2.5. Sampling and analysis of MBTs

Groundwater samples (500 mL to 1 L of groundwater) were collected from Sites 1 and 2 to supplement contaminant and geochemical data to characterize microbiological conditions and potential contaminant biodegradation processes. Groundwater samples for molecular analyses were collected concurrently with contaminant and geochemistry samples from monitoring wells within impacted areas, including source and downgradient plume areas, and from background monitoring well locations for comparison. Groundwater samples from Sites 1 and 2 were shipped overnight on ice to Microbial Insights, Inc. of Knoxville, TN (MI). MI personnel extracted DNA from the groundwater samples.

qPCR was performed on the Site 1 samples for PCR primers targeting the *Dehalococcoides* (DHC) 16S rRNA gene, a genus capable of complete, sequential reductive dechlorination of TCE (and tetrachloroethene) to the non-toxic end product ethene (Maymó-Gatell et al., 1997; Löffler et al., 2013). DHC obtain metabolic energy by using hydrogen as an electron donor, acetate as a carbon source, and halogenated organic compounds, such as TCE, as electron acceptors. MI performed qPCR utilizing assay details, primers, and thermocycler conditions as previously described (Hatt et al., 2013).

Due to limited dissolved oxygen present in groundwater (i.e., <1 mg/L), qPCR analysis was performed on the Site 2 samples to detect and quantify functional genes related to anaerobic PHC biodegradation mechanisms including the benzoyl coenzyme A reductase (*bcrA*) gene implicated in cleavage of the benzene ring structure under anaerobic conditions (Boll and Fuchs, 1995), benzylsuccinate synthase (*bssA*) gene implicated in anaerobic biodegradation of toluene and xylenes (Biegert et al., 1996; Achong et al., 2001; Beller et al., 2002; Heider et al., 2016; Rabus et al., 2016), naphthyl-2-methyl-succinate synthase (*mnssA*) gene (Selesi et al., 2010), anaerobic naphthalene carboxylase genes (ANC; Mouttaki et al., 2012), and alkylsuccinate synthase (*assA*) which is linked to anaerobic biodegradation of alkane PHCs. Further, the adenosine-5′-phosphosulfate reductase gene (APS) characteristic of sulfate-reducing bacteria (SRB; Friedrich, 2002), which can be associated with anaerobic PHC biodegradation was also quantified (Johann Heider et al., 1998). MI performed qPCR utilizing commercially available assays for *bssA*, *assA*, *mnssA*, *bcrA*, ANC, and APS. It is important to note that some of the commercially available assays involve the use of multiple qPCR assays (five assays for *bssA*, six for *assA*, two for *mnssA*, three for *bcrA*, and seven for APS) for which quantities are added as previously described (Beller et al., 2002; Rossmassler et al., 2019; Kharey et al., 2020).

All qPCR analyses were performed using TaqMan™ 2X Universal PCR Master Mix (Thermo Fisher Scientific™) and were run on the QuantStudio™ 12 K Flex (Thermo Fisher Scientific™) with a thermal cycling amplification profile for including a single cycle of 2 min at 50°C for uracil-N-glycosylase (UNG) enzyme activation, followed by a single cycle of 10 min at 95°C to inactivate UNG enzyme, template DNA denaturing, and AmpliTaq Gold polymerase activation, followed by 40 cycles of 15 s at 95°C and 1 min at 60.0°C for repeated denaturing, polymerase annealing, and extension (Hatt et al., 2013). qPCR data herein are reported as cells/mL, which were calculated by dividing gene copies/mL by the number of gene copies/genome and dividing by the number of genomes/cell. All gene targets analyzed in this manuscript were assumed to have one genome per cell and one gene copy per genome.

Additionally, taxonomic 16S rRNA gene sequencing (i.e., NGS) was performed to assess microbial community composition and potential phyla and genera linked to PHC biodegradation. MI performed NGS of the 16S rRNA gene utilizing MiSeq Reagent Kit V3 300 cycle (Illumina^®^) and Nextera Index Kit V2 Set A (Illumina^®^) and were run on the MiSeq (Illumina^®^). Universal primers targeting the V3 and V4 regions of bacterial 16S rRNA genes were used as previously described (Klindworth et al., 2013): forward primer (S-D-Bact-0341-b-S-17) 5′-CCTACGGGNGGCWGCAG and reverse primer (S-D-Bact-0785-a-A-21) 5′-GACTACHVGGGTATCTAATCC. Illumina adapter overhang nucleotide sequences were added to the primer sequences and NGS library preparation methods were followed as described in the 16S Metagenomic Sequencing Library Preparation Protocol (Illumina^®^).

It should be noted that while qPCR and NGS were applied at these two sites to support site objectives, additional MBTs (e.g., metabolomics or proteomics) may have been useful to assess and demonstrate *in situ* contaminant biodegradation and support site decision-making.

## 3. Results

### 3.1. Site 1: TCE bioremediation

The field-scale framework was applied and SSBOs were established to assess the potential for natural or enhanced bioremediation of the primary contaminant, TCE. Baseline chemical characterization as part of the initial step of the framework process was performed and demonstrated that TCE and the first reductive dechlorination daughter product, cis-1,2-dichloroethene (cDCE), were the dominant contaminants present in saturated soil (TCE and cDCE maxima of 3,970 μg/kg and 364 μg/kg respectively) and groundwater. Vinyl chloride (the second daughter product of sequential reductive dechlorination of TCE) and non-toxic end products, ethene and ethane, were generally absent in groundwater suggesting that complete reductive dechlorination of TCE was not occurring (De Bruin et al., 1992; Maymó-Gatell et al., 1997; Wiedemeier et al., 1999; Amos et al., 2008). Since TCE can be degraded by natural dechlorinating microorganisms that utilize hydrogen, formate, or other fermentation products as an electron donor, it was hypothesized that contaminant-degrading activities of native dechlorinating microorganisms were limited by the availability of fermentable substrates or electron donor (i.e., organic carbon) and/or that these microorganisms were not present in sufficient abundance (Sung et al., 2006; Moe et al., 2009; Löffler et al., 2013; Key et al., 2017; Yang et al., 2017; Chen et al., 2022; Jiang et al., 2022). Based on these observations, additional evaluation was performed in accordance with a standardized framework to assess the feasibility of an *in situ* biostimulation strategy to treat TCE and lesser chlorinated daughter products.

#### 3.1.1. Assessment

Initial baseline geochemical characterization in November 2010 indicated that while plume conditions were anaerobic as shown by the absence of dissolved oxygen in groundwater monitoring wells, the geochemical conditions were not favorable for intrinsic reductive dechlorination (Table 1). Detections of electron acceptors nitrate (up to ~40 mg/L) and sulfate (~100 mg/L) in conjunction with low levels of organic carbon (TOC <10 mg/L) indicated limited intrinsic microbial fermentation activities needed to produce hydrogen, formate, and similar electron donor substrates utilized by DHC and other microorganisms during reductive dechlorination (Sung et al., 2006; Moe et al., 2009; Löffler et al., 2013; Key et al., 2017; Yang et al., 2017; Chen et al., 2022; Jiang et al., 2022). Groundwater samples collected for qPCR analysis confirmed that DHC were present, but at abundances [10^1^–10^3^ cells per milliliter (cells/mL); detection limit of 10^−1^ cells/mL] that likely limited the rate of biodegradation. Therefore, while the requisite dechlorinating bacteria were present demonstrating that a bioremediation approach was feasible, the data indicated that biostimulation could be a useful strategy to increase the availability of fermentable substrates and promote geochemical conditions favorable for sustained *in situ* bioremediation through reductive dechlorination.

**Table 1:** Summary of baseline ranges for key parameters measured during the Assessment Stage from groundwater monitoring wells within the plume core.

Parameter	Shallow zone	Intermediate zone	Deeper zone
*Parent contaminant and associated daughter products*			
TCE	1,490–3,900 μg/L	148–25,400 μg/L	2,660–28,800 μg/L
cDCE	682–1,140 μg/L	54.8–4,450 μg/L	5,410 μg/L
Vinyl chloride	<1–25 μg/L	<1–1.6 μg/L	<1 μg/L
Ethene	<10 μg/L	<10 μg/L	<10 μg/L
Ethane	<10 μg/L	<10 μg/L	<10 μg/L
*Geochemical indicator parameters*			
TOC	0.5–4.1 mg/L	<1–3.6 mg/L	<1–2.6 mg/L
Dissolved oxygen	<1 mg/L	<1 mg/L	<1 mg/L
Nitrate	25.2–40.6 mg/L	<0.1 mg/L	<0.1–0.17 mg/L
Sulfate	87–106 mg/L	52.3–122 mg/L	65.4–68.3 mg/L
Methane	<10 μg/L	<10 μg/L	<10 μg/L
*MBT (qPCR analysis for 16S rRNA gene)*			
DHC	1.5 × 10^2^–1.85 × 10^3^ cells/mL	6.2 × 10^2^–9.8 × 10^2^ cells/mL	1.2 × 10^1^–4.3 × 10^2^ cells/mL

#### 3.1.2. Design

The contaminant, geochemical, and MBT data generated during the Assessment Stage provided MLOE that a biostimulation approach to enhance intrinsic bioremediation could be effective. The Assessment Stage results indicated that low levels of natural organic carbon in the source area and associated groundwater plume likely limited the intrinsic reductive dechlorination capacity of native dechlorinating microorganisms. Therefore, based on these data and hydrogeologic understanding, injection of a soluble organic carbon amendment (sodium lactate solution) was posited to be effective in enhancing intrinsic reductive dechlorination activities. During the Design Stage, the mass of lactate in the amendment solution (25 kg of lactate in 750 gallons of water) was calculated to provide the electron equivalents necessary to satisfy the demand for reductive dechlorination of chlorinated contaminants in aqueous and sorbed phases (assuming equilibrium partitioning to aquifer solids) and other competing electron acceptors in the system (e.g., nitrate, iron and manganese oxides, and sulfate) over a period of four injection events (Supplementary Table S1).

**Figure 3:**
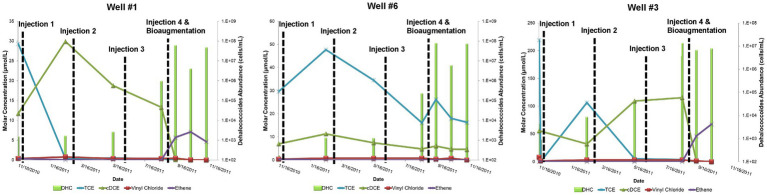
Site 1 field pilot study performance monitoring trends for TCE, cDCE, vinyl chloride, ethene, and DHC.

During the Design Stage, a pilot test was conducted that consisted of four, quarterly, low-pressure (<20 pounds per square inch of injection backpressure) amendment injections (25 kg lactate in 750 gallons of water) in three injection wells that targeted the shallow and intermediate portion of the plume nearest the source area between November 2010 and August 2011. Pilot performance groundwater samples for contaminant chemistry, geochemical indicator parameters, and MBTs (i.e., qPCR for DHC abundance) were collected from four groundwater monitoring wells (Well #1, Well #3, Well #7, and Well #6) ~2 months after each injection. With the exception of Well #7, which was determined to be outside of the radius of influence of injections, TCE concentrations decreased following the first injection and remained <50 μg/L in pilot wells following the second and third injections (Figure 3). Conversely, levels of cDCE varied but remained elevated following the first three pilot injection events indicating stalled reductive dechlorination (sometimes referred to as “cDCE stall”). This was further evidenced by the absence of corresponding increases in vinyl chloride or ethene concentrations. Despite positive influence in the treatment area including increased TOC levels and redox transition to sulfate-reducing conditions (i.e., decreased sulfate levels) that promote reductive dechlorination processes (Wiedemeier et al., 1999), the amendment had modest effects on the abundance of the native DHC population capable of complete reductive dechlorination (Figure 3). The limited effect on the DHC population may have been due to competition for growth substrates with other native microorganisms, which was not evaluated. These contaminant and MBT data supported the decision to bioaugment with a commercially available bioaugmentation DHC culture (SDC-9™ comprising more than 10^7^ DHC cells/mL), which was added to the three pilot injection wells (2 L per well) during the fourth quarterly amendment injection event. DHC abundance increased by up to three orders of magnitude (OoM) after the fourth injection event (10^5^–10^8^ cells/mL) and cDCE concentrations decreased while the concentration of ethene increased (Figure 3).

The pilot test results during the Design Stage were used to conclude that (1) injection of a lactate amendment could favorably achieve biogeochemical conditions that promote reductive dechlorination, and (2) biostimulation was unlikely to stimulate growth of native DHC to support complete reductive dechlorination at the site and bioaugmentation was warranted following establishment of appropriate, reducing geochemical conditions.

#### 3.1.3. Performance monitoring

**Figure 4:**
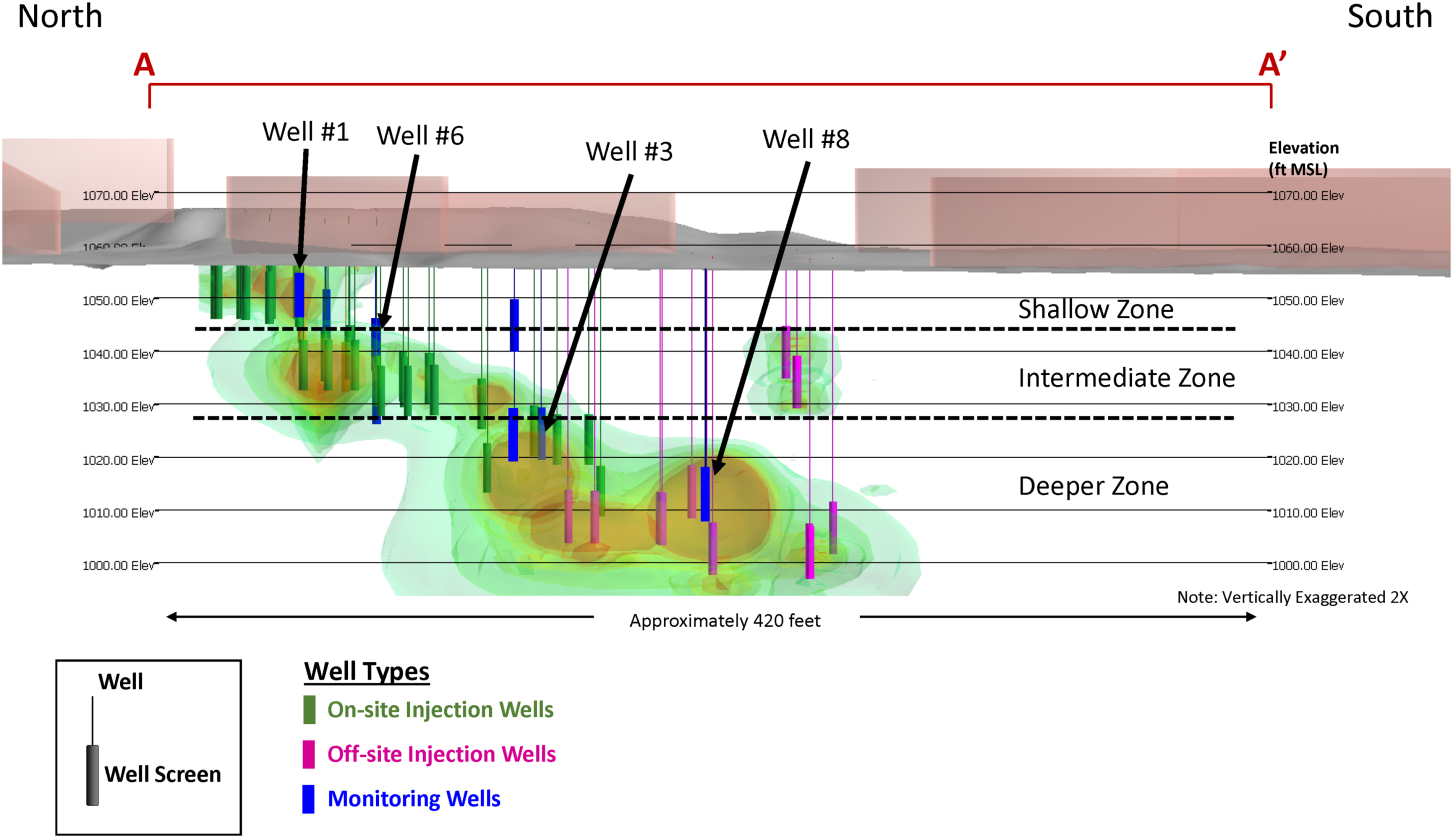
Site 1 cross-sectional view of full-scale implementation of the enhanced bioremediation strategy.

Following and informed by the pilot test, an expanded amendment injection program was designed, and implemented with 31 additional on-site and off-site injection wells in the shallow source area and the intermediate and deep hydrostratigraphic zones of the dissolved plume (Figure 4). Amendment injections were performed on a quarterly basis between October 2012 and November 2014 in the intermediate zone and deeper zone using the lactate dosage from the pilot test of 25 kg of lactate in 750 gallons of water. Amendment injections were performed in October 2012 and January 2013 in the shallow zone and were discontinued due to attainment of regulatory criteria for TCE, cDCE, and vinyl chloride.

**Figure 5:**
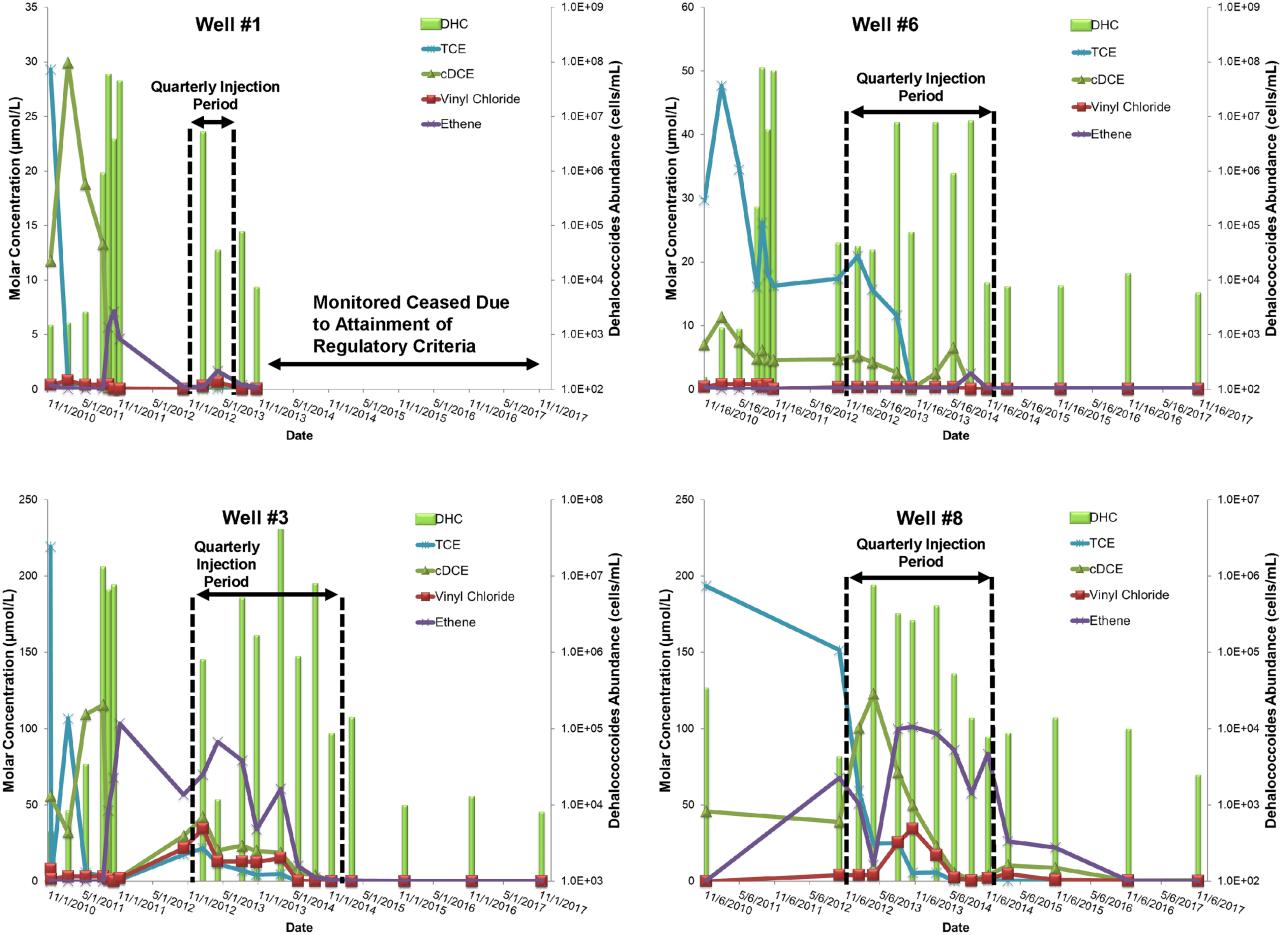
Site 1 full-scale implementation of enhanced bioremediation performance monitoring trends of TCE, cDCE, vinyl chloride, ethene, and DHC within the treatment area.

Similar to the pilot test, groundwater samples were collected from nine on-site and off-site monitoring wells ~1 month prior to each amendment injection event for contaminant chemistry, geochemical indicator parameters, and MBT data (DHC abundances). Overall, the results of the full-scale amendment injection program mirrored the results of the pilot test. The amendment injections increased the availability of organic carbon (TOC concentrations >100 mg/L in remedy performance monitoring wells) and promoted geochemically reducing conditions favorable for reductive dechlorination. Following the first several on-site and off-site amendment injection events, TCE concentrations within the dissolved plume in the hydrostratigraphic zones decreased (Figure 5). Consistent with the pilot test, the levels of cDCE remained elevated in the intermediate and deeper zones outside of the pilot test treatment zones. The qPCR results showed that the abundance of DHC did not substantially increase in the intermediate and deeper treatment zones, consistent with the observations of incomplete reductive dechlorination.

Bioaugmentation with the commercially available SDC-9™ DHC culture was added to the injection wells screened in the intermediate and deep treatment zones (2 L per injection well) during the April 2013 amendment injection event. Following bioaugmentation, abundances of DHC increased up to four and three OoM, respectively, to 10^3^–10^7^ cells/mL in the intermediate zone and 10^3^–10^6^ cells/mL in the deep zone. cDCE levels generally decreased in the June 2013 groundwater sampling event in both intermediate and deep zones, while vinyl chloride and ethene also increased above baseline levels. Continued quarterly amendment injection continued until November 2014 and TCE, cDCE, and vinyl chloride levels decreased below baseline levels across the plume. Based on attainment of regulatory criteria across a substantial portion of the plume, the bioremediation strategy was shifted in March 2015 to a MNA strategy for a 3-year period to assess the long-term stability of the treatment area. The lack of plume rebound except for two isolated areas and the continued detection of DHC indicated that natural biodegradation capacity was sufficient to meet remedial and regulatory objectives and the site was transitioned to a long-term monitoring regulatory program.

#### 3.1.4. Key lessons

Application of the field-scale framework at the Assessment Stage demonstrated that intrinsic reductive dechlorination was occurring at the site, but that the activities of native dechlorinating bacteria were limited by electron donor availability. Field pilot studies during the Design Stage demonstrated that injection of an organic carbon amendment could modify geochemical conditions to those that favor reductive dechlorination, but the MBT data (i.e., qPCR results) demonstrated that abundance of native populations of DHC did not substantially increase suggesting that bioaugmentation should be part of the remedial strategy at this site. Following full-scale implementation of the enhanced bioremediation strategy, groundwater monitoring during the Performance Monitoring Stage demonstrated remedy success through a MLOE approach, including decreasing plume contaminant concentrations (primary line of evidence), shift toward reducing geochemical conditions favorable for reductive dechlorination (secondary line of evidence), and increased DHC abundance following bioaugmentation and injections sufficient for enhanced bioremediation as determined by MBT analyses, specifically qPCR (tertiary line of evidence).

### 3.2. Site 2: PHC bioremediation

The field-scale framework was applied to the Site and SSBOs were established to assess the potential intrinsic biodegradation capacity to assess whether bioremediation was applicable to mitigate Site contamination. As the initial step of the framework application, critical review of historical groundwater data collected between August 2010 and March 2018 demonstrated that plume contraction had occurred during this period within the source areas and in the downgradient plume (Figure 2). Due to the observed TPH-Dx plume contraction, coincident absence of TPH-Gx and BTEX outside the source area, and ability for PHC biodegradation by natural microorganisms that utilize oxygen, sulfate, or other terminal electron acceptors, it was hypothesized that (i) intrinsic contaminant degradation was occurring and (ii) contaminant-degrading activities of native microorganisms are limited by the availability of electron acceptor (i.e., sulfate). Therefore, an additional SSBO was established and a study was implemented to assess the feasibility of intrinsic bioremediation and/or enhanced bioremediation as a potential component(s) of the site management strategy.

#### 3.2.1. Assessment stage

**Figure 6:**
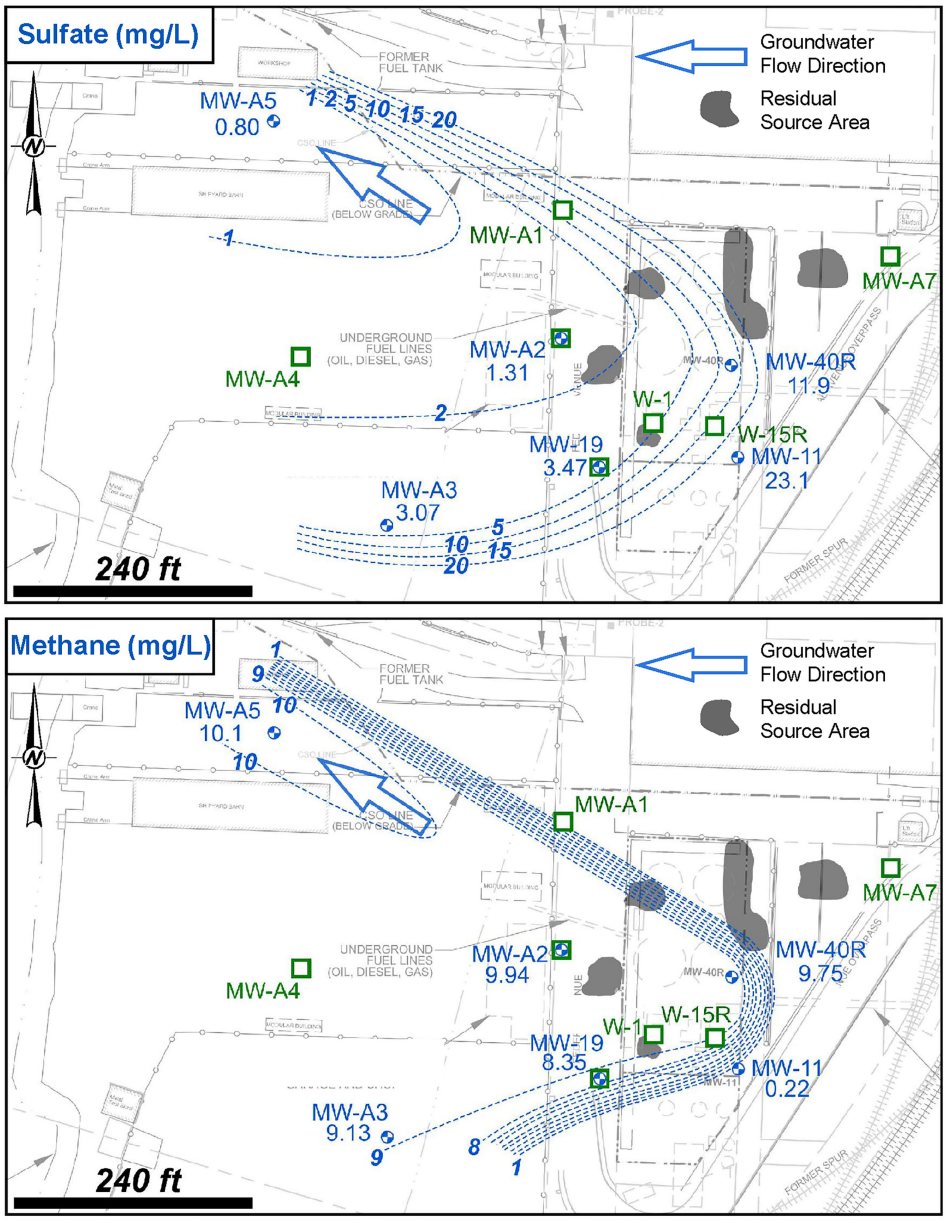
Site 2 sulfate (mg/L) and dissolved methane (mg/L) distribution in shallow groundwater as determined during the Assessment Stage.

Analytical results of geochemical parameters indicated that anaerobic and strongly reducing biogeochemical conditions were present in the on-site source area, as monitored by W-1, W-15R, and MW-A1, and extend into the downgradient plume area, as monitored by MW-19, MW-A2, and MW-A4. Spatial distribution plots of key biogeochemical indicator parameters sulfate and dissolved methane are presented in Figure 6. Review of these data in comparison to background monitoring well MW-11 showed a depletion of sulfate and concomitant increase in dissolved methane in groundwater monitoring wells that correlate to decreased TPH-Dx concentration with increasing distance from the source area. This is a well-characterized condition of PHC sources and plumes where the excess availability of PHC (and potentially other organic compounds), which are microbial growth substrates and/or electron donors, promotes these anaerobic and strongly reducing geochemical conditions (Wiedemeier et al., 1999; Roychoudhury and McCormick, 2006; Suthersan et al., 2011). Overall, the depletion of sulfate below the background and increased dissolved methane levels above the background suggested that intrinsic biodegradation of TPH-Dx was occurring by both sulfate reduction and methanogenesis pathways, as well as by fermentation processes.

**Figure 7:**
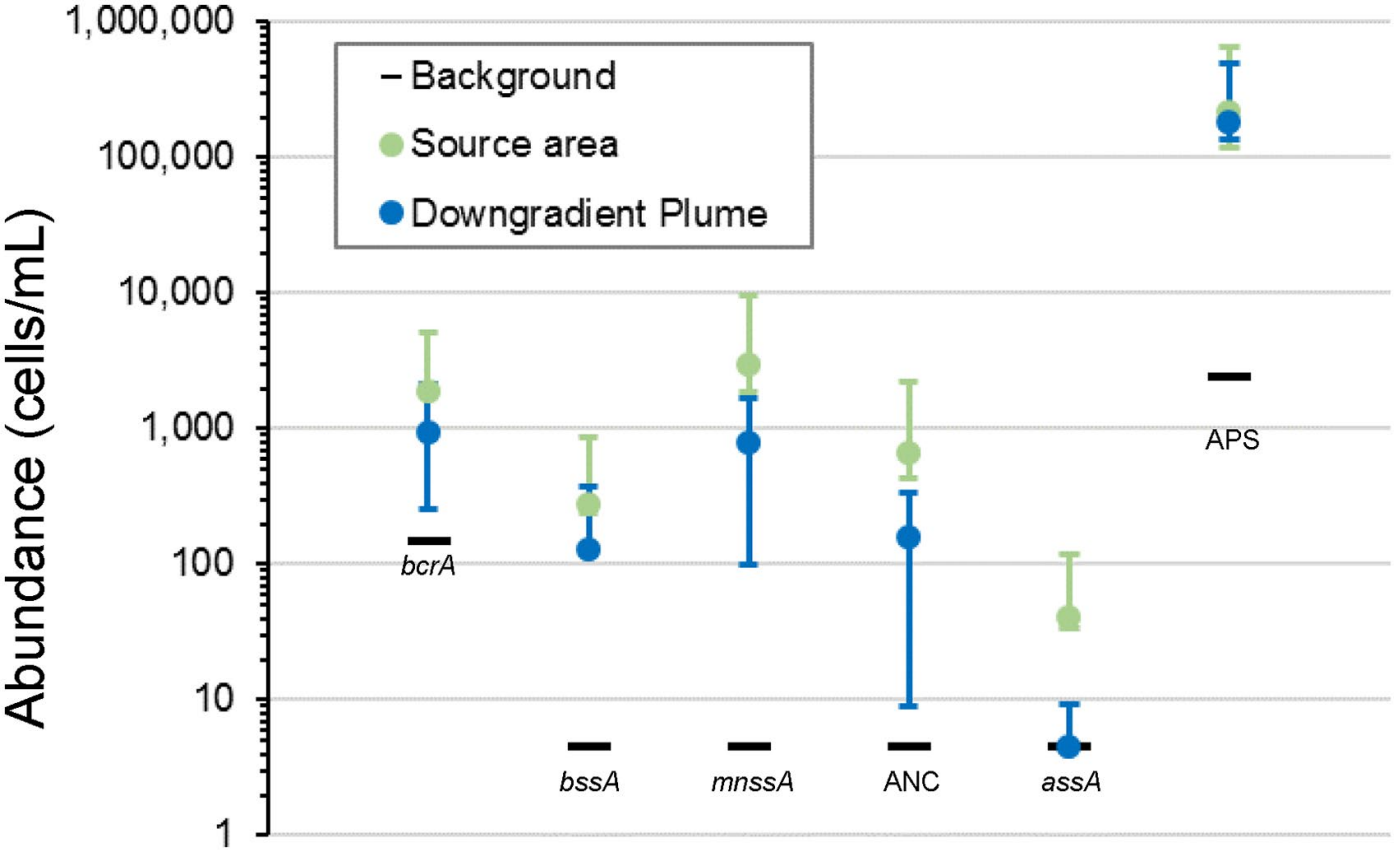
Site 2 abundance of functional genes in background, source area, and downgradient plume monitoring wells related to anaerobic degradation of PHC. The mean abundance for functional genes in source area and downgradient plume monitoring wells is represented by the green and blue circles, respectively, and the data spread (maximum and minimum) is represented by the error bars. The measured abundance for functional genes in the background monitoring well is represented by the black horizontal bars. Abundances of functional genes related to PHC biodegradation in source area and downgradient plume monitoring wells are several OoM greater than abundances in the background monitoring well.

qPCR analyses (Figure 7) showed that the abundance of functional genes related to anaerobic degradation of TPH-Gx, TPH-Dx, and BTEX in the source area and downgradient plume was as high as three OoM greater than the abundance in background wells. The benzoyl coenzyme A reductase (*bcrA*) gene implicated in cleavage of the benzene ring structure under anaerobic conditions (Boll and Fuchs, 1995) and benzylsuccinate synthase (*bssA*) gene implicated in anaerobic biodegradation of toluene and xylenes (Biegert et al., 1996; Achong et al., 2001; Beller et al., 2002; J Heider et al., 2016; Rabus et al., 2016) were detected in source area monitoring wells (*bcrA*: 9.5 × 10^2^–1.5 × 10^3^ cells/mL; *bssA*: 4.4 × 10^1^–2 × 10^2^ cells/mL) and downgradient plume area monitoring wells (*bcrA*: 3.2 × 10^2^–3.2 × 10^3^ cells/mL; *bssA*: not detected to 6 × 10^2^ cells/mL), which were at higher abundances compared to the background area well (*bcrA*: 1.5 × 10^2^ cells/mL; *bssA*: not detected). Abundances of functional genes associated with anaerobic biodegradation of TPH-Dx components, including naphthyl-2-methyl-succinate synthase (*mnssA*) gene (Selesi et al., 2010) and anaerobic naphthalene carboxylase genes (ANC; Mouttaki et al., 2012) were also as high as two OoM higher in source area (*mnssA*: 1.1 × 10^3^–1.2 × 10^3^ cells/mL; ANC: 2.4 × 10^2^–2.7 × 10^2^ cells/mL) and downgradient plume monitoring wells (*mnssA*: not detected to 6.7 × 10^3^ cells/mL; ANC: not detected to 1.5 × 10^3^ cells/mL) as compared to background (*mnssA* and ANC not detected above detection limits of 4.6 cells/mL). The abundance of alkylsuccinate synthase (*assA*) which is linked to anaerobic biodegradation of alkane PHCs was also elevated in the source area and plume samples (not detected to 5.2 × 10^2^ cells/mL) as compared to background. Further, many SRB, detected and measured by APS (Ramos et al., 2012), anaerobically degrade PHCs (Heider et al., 1998). The abundance of APS in the source area and downgradient plume monitoring wells are nearly two OoM greater than background, suggesting SRB as relevant PHC-degrading microorganisms at the site.

**Figure 8:**
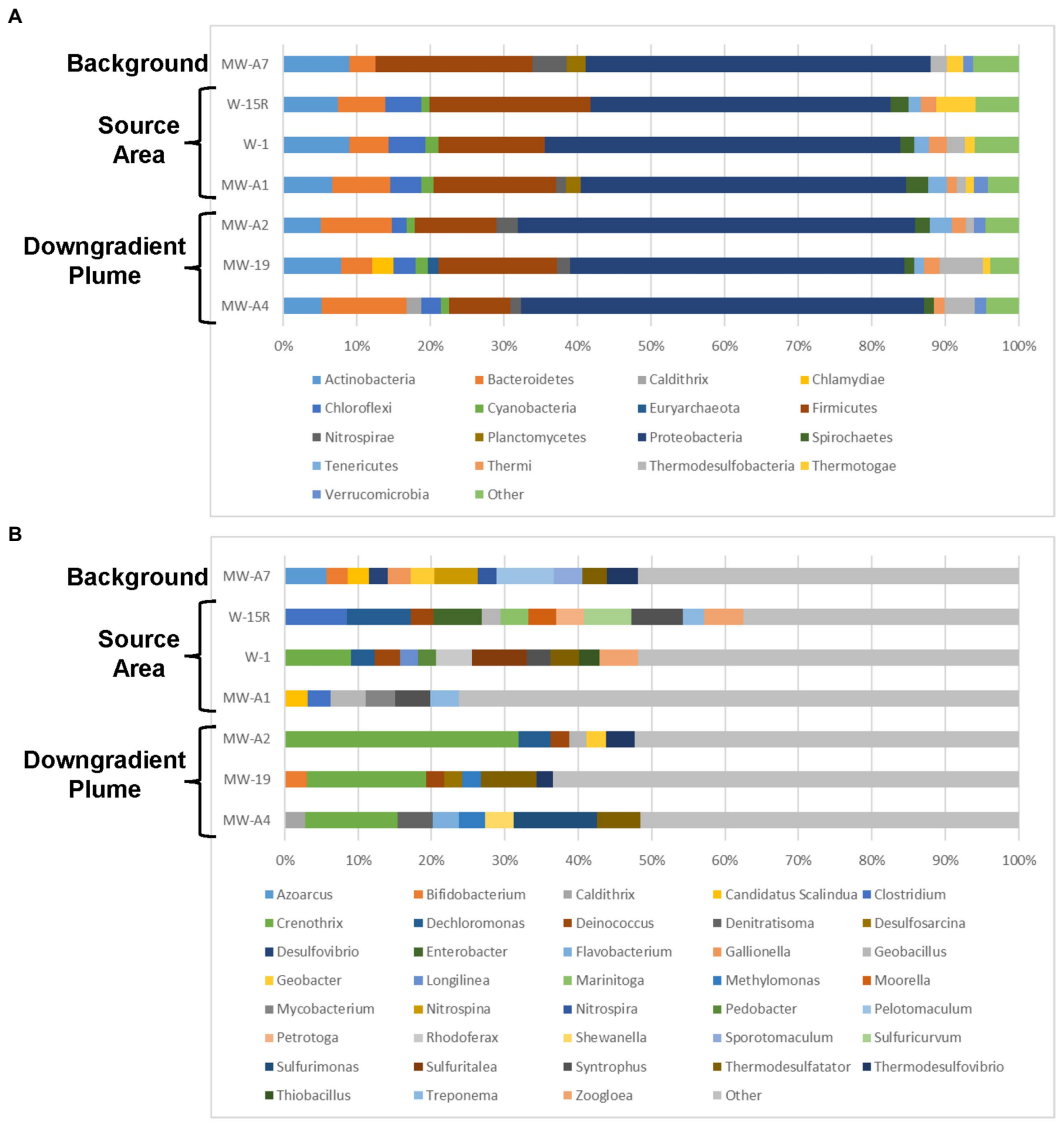
Site 2 taxonomic distribution of bacterial communities for each sample location. Operational taxonomic units (OTUs) detected **(A)** at the phylum level (>1% of relative abundance) and **(B)** at the genus level (>2% relative abundance).

The 16S rRNA gene NGS results identified that changes in microbial community composition and relative abundance are apparent in source and downgradient plume areas as compared to background (Figure 8). Several genera known to be implicated in biodegradation of PHCs and various TPH constituents are found above 2% relative abundance in samples from source area (primarily W-15R, W-1, and MW-A1) and downgradient plume (MW-A2) monitoring wells that are absent from the background sample. Genera detected in source area and plume area monitoring wells above background relative abundances and linked to PHC biodegradation include *Dechloromonas* (Chakraborty et al., 2005), *Deinococcus* (Liang et al., 2011), *Clostridium* (Gieg et al., 2014), *Geobacillus* (Elumalai et al., 2019), *Syntrophus* (Gieg and Toth, 2019), *Zoogloea* (Farkas et al., 2015), *Enterobacter* (Ramasamy et al., 2017), and *Petrotoga* (Purwasena et al., 2014). In addition to the presence of PHC degraders in the source and plume area, methanogenic microorganisms including those belonging to the Classes *Methanobacteria* and *Methanomicrobia* were also found in higher relative abundance in the source and downgradient plume areas above the background suggesting hydrocarbon degradation to methane by methanogens is likely occurring.

#### 3.2.2. Design

The contaminant, geochemical, and MBT data generated during the Assessment Stage provide strong evidence to suggest that intrinsic, anaerobic biodegradation processes are supporting contaminant attenuation and temporal plume contraction within the source and downgradient plume areas. Decreasing TPH-Dx and TPH-Gx concentration trends for plume and source area groundwater monitoring wells (Supplementary Figure S1) and associated plume contraction (Figure 2), most significant within the downgradient portion, are primary lines of evidence of contaminant attenuation and potential biodegradation. Geochemical characterization provides a secondary line of evidence of biodegradation. Depletion of sulfate below the background concomitant with increasing dissolved methane levels in the source area infers contaminant biodegradation is occurring by sulfate reduction, methanogenesis, and fermentation pathways. MBT results (qPCR and NGS data) provide a tertiary line of evidence of PHC biodegradation as functional genes and several genera known to be implicated in PHC biodegradation were observed in higher relative abundance in the source and downgradient plume as compared to background.

Overall, the Assessment Stage data provide sufficient, strong evidence that supports occurrence of intrinsic bioremediation across the source area and downgradient groundwater plume. Given the collapse of the downgradient TPH-Dx plume to levels near regulatory criteria and demonstrated intrinsic biodegradation, a MNA approach was determined to be appropriate for the downgradient plume. A monitoring program developed during the Design Stage that includes continued sampling of downgradient plume monitoring wells (MW-A2, MW-A4, MW-A5, MW-A6, and MW-19) for analysis for contaminants, key geochemical indicator parameters (e.g., sulfate, dissolved methane), and key functional and taxonomic genes related to anaerobic biodegradation of PHCs (*bcrA*, *bssA*, *mnssA*, ANC, *assA*, APS) was recommended for performance monitoring of MNA of the downgradient plume.

**Figure 9:**
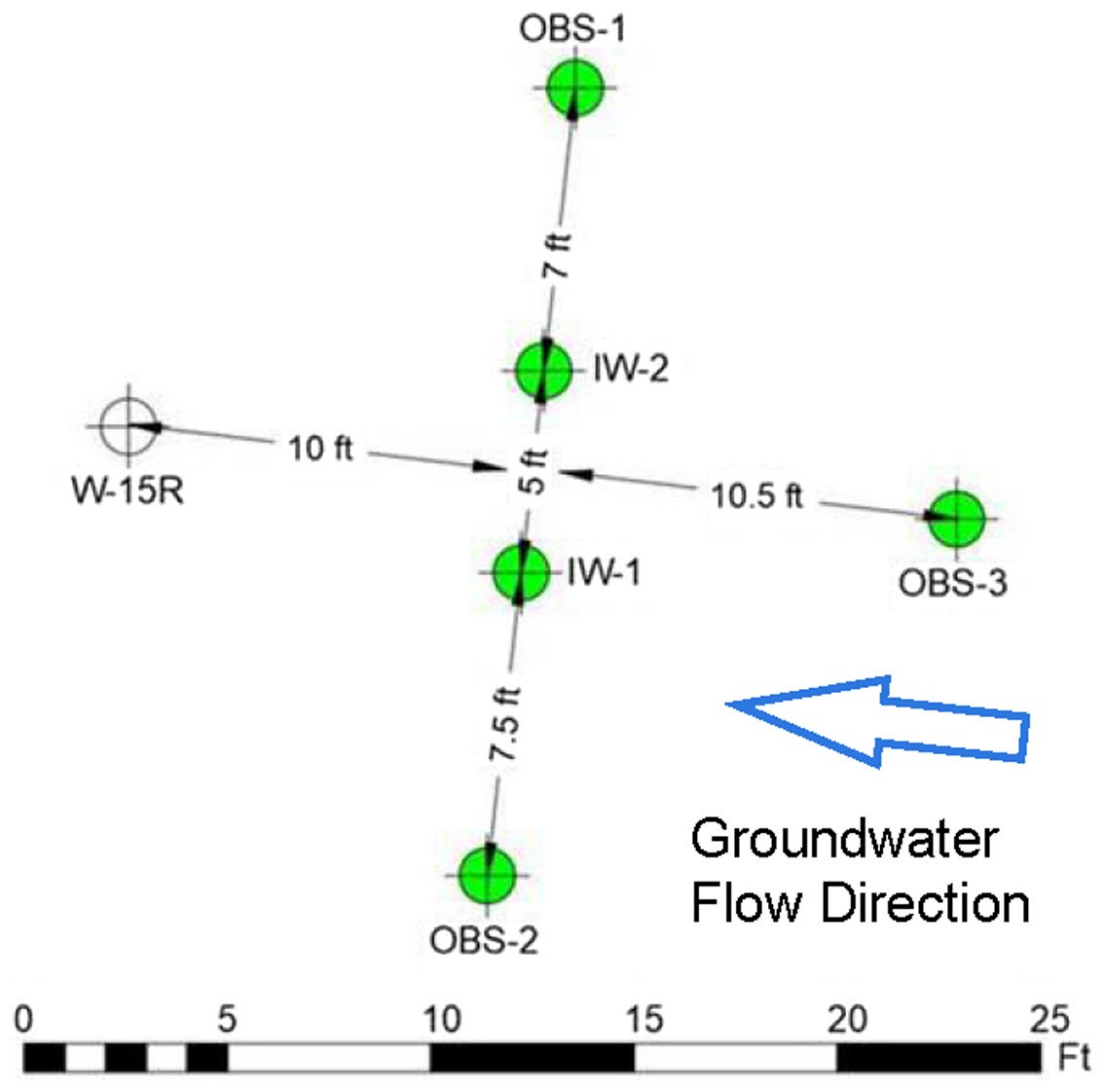
Site 2 field pilot study layout. IW-1 and IW-2 are injection wells. OBS-3 monitors conditions upgradient of the injection area. OBS-1 and OBS-2 monitor conditions cross-gradient to the injection area. W-15R monitors conditions downgradient of the injection area.

While the data generated during the Assessment Stage showed that source area TPH biodegradation is occurring, the depletion of electron acceptors (primarily sulfate) is potentially limiting the biodegradation capacity of the residual PHCs in the source areas (Anderson and Lovley, 2000; Sublette et al., 2006; Buscheck et al., 2019). Therefore, a field pilot study was designed to assess whether injection of a sulfate amendment (i.e., soluble magnesium sulfate solution) and nitrogen and phosphorous nutrients (diammonium phosphate) could promote native SRB activities to enhance intrinsic biodegradation of PHCs. The pilot study location was selected in the area immediate to W-15R based on observations of consistently elevated TPH-Dx aqueous concentrations in shallow groundwater. The pilot study comprised two injection wells (IW-1 and IW-2) sited 10 feet upgradient of W-15R, and four performance monitoring wells (OBS-1, OBS-2, OBS-3, and W-15R) as shown in Figure 9. The initial amendment was designed to comprise 15 kg of magnesium sulfate in 500 gallons of potable water, which was estimated to target a sulfate concentration of ~100 mg/L across a treatment area with a thickness and radius of 10 ft. and 20 ft., respectively. In addition to baseline sampling, performance groundwater sampling events were designed to be conducted monthly after the initial injection and then quarterly for the duration of the 1-year study.

#### 3.2.3. Performance monitoring

**Figure 10:**
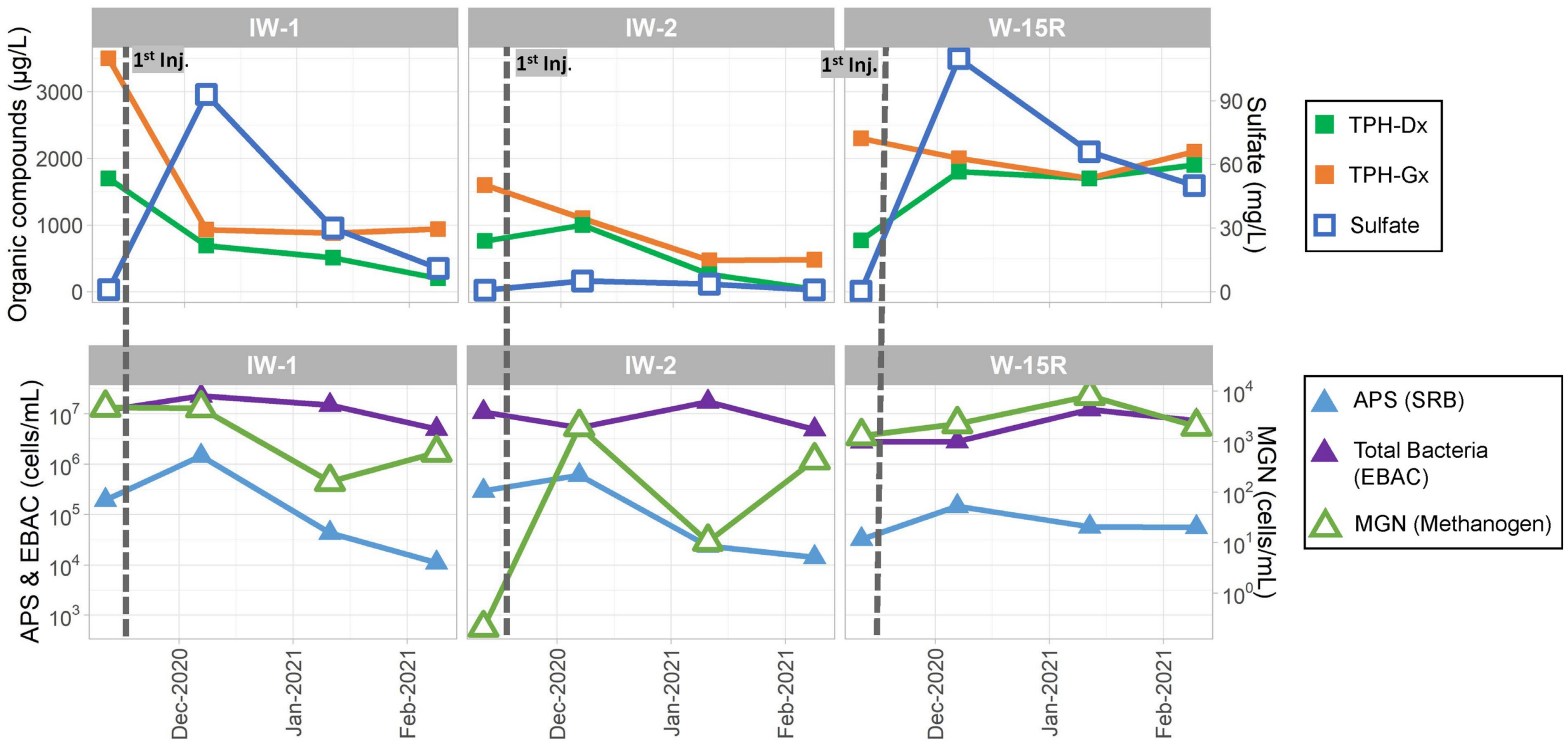
Site 2 field pilot study performance monitoring results for the injection wells (IW-1 and IW-2) and the downgradient well (W-15R) before and 3 months after the initial November 2021 injection.

The sulfate amendment was initially injected in November 2020. One, two, and three months (December 2020, January 2021, and February 2021 respectively) after injection, performance groundwater samples were collected for analyses of contaminants (TPH-Dx and TPH-Gx), geochemical parameters, and MBTs (qPCR targeting genes for SRB, methanogens, and total bacteria) to assess the initial effects of the sulfate amendment on native microorganisms and associated anaerobic biodegradation of PHCs. As shown in the concentration trends presented in Figure 10, following injection in November 2020, sulfate levels initially increased from baseline concentrations of <1 mg/L to near 100 mg/L in December 2020 in IW-1 and W-15R, and to 45 mg/L in OBS-2 and then declined during January and February 2021 toward baseline presumed to be due to a variety of attenuation mechanisms, including consumption by native SRBs, advection, and dilution. This trend was mirrored with the qPCR data measuring the APS gene abundance to monitor SRB. APS abundances increased in IW-2, IW-3, and W-15R in December 2020 and then declined thereafter concomitant with decreasing sulfate availability. These lines of evidence suggest the injected sulfate had effectively biostimulated native SRB to promote biodegradation activity within the immediate injection area. This observation was further supported by the observed decreases of TPH-Dx and TPH-Gx during the initial 3 months in IW-1, IW-2, and OBS-2. However, the limited or absent sustained reduction in contaminant levels outside of the immediate injection (IW-1) and downgradient areas (W-15R) suggests that the distribution of sulfate and biostimulation across the broader treatment area was limited.

The sulfate dosage was subsequently increased to 30 kg of magnesium sulfate in 500 gallons of water for the second (February 2021) and third (May 2021) injection events. Owing to increased sulfate availability, abundance of SRBs (measured by APS) increased by more than one OoM in April 2021 as compared to February 2021 in IW-1 (1.11 × 10^4^ cells/mL to 6.53 × 10^5^ cells/mL), IW-2 (1.41 × 10^4^ cells/mL to 2.09 × 10^5^ cells/mL), OBS-2 (6.34 × 10^3^ cells/mL to 2.23 × 10^5^ cells/mL), and W-15R (5.59 × 10^4^ cells/mL to 2.60 × 10^6^ cells/mL) as shown in Supplementary Figures S2,S3. While levels of TPH-Dx and TPH-Gx remained below baseline levels in IW-1, IW-2, and OBS-3, TPH-Dx and TPH-Gx concentrations in samples from W-15R were variable with consistent concentrations above 1,000 μg/L detected. The elevated detection of TPH-Dx above baseline levels in IW-1 in July 2021 is considered likely anomalous due to the absence of concomitant increase of TPH-Gx. The variability of TPH-Dx observed at W-15R is consistent with the variability measured in upgradient well OBS-3 and cross-gradient well OBS-1 (Supplementary Figure S3), thus the influence of injection effects cannot be determined based on aqueous TPH-Dx concentrations as a single line of evidence. However, additional lines of evidence including increased followed by decreased sulfate concentrations with concomitant increase followed by decrease in APS abundance after the February 2021 and May 2021 injections at W-15R suggest that biodegradation of PHCs was enhanced through biostimulation using sulfate as an amendment. The presence of residual source material in soil may obscure potential positive effects of enhanced bioremediation observable by aqueous phase TPH concentrations due to increased rate of dissolution or desorption of source material as multi-phase equilibrium is maintained (Seagren et al., 1994; Amos et al., 2008). Further, it did not go unnoticed that analysis of additional functional genes *via* qPCR, such as alkane succinate synthase (*assA*), or application of additional MBTs (e.g., metabolomics) may have provided further evidence to assess effectiveness of enhanced bioremediation of TPH constituents through biostimulation with sulfate.

Overall, the results of the field pilot performance monitoring demonstrated through MLOE including contaminant, geochemistry, and MBT (i.e., qPCR) suggest that direct injection of a sulfate amendment could promote growth, increased abundance, and presumably activity of PHC-degrading SRBs. However, while SRB abundance did increase above pre-injection levels downgradient of the injection area suggesting increased biodegradation capacity, TPH-Dx and TPH-Gx concentration trends did not exhibit sustained decreasing concentration trends. These data suggest that a higher sulfate mass dosage and potentially a less soluble sulfate source, such as gypsum, to sustain elevated levels of sulfate for longer durations should be considered to enhance bioremediation capacity and activity at the site.

#### 3.2.4. Key lessons

Application of the field-scale framework at the Assessment Stage demonstrated that intrinsic biodegradation of the PHCs was occurring within the source and downgradient plume areas. MLOE including contaminant spatiotemporal trends, geochemical characterization, and abundance of functional genes and microorganism linked to anaerobic PHC biodegradation above background abundances provided strong evidence that groundwater remedial objectives could be achieved by natural intrinsic bioremediation. Yet, the data suggested that limited availability of electron acceptors may be restricting intrinsic bioremediation in the source area. The field pilot study developed during the Design Stage demonstrated during the Performance Monitoring Stage that a sulfate amendment could promote increased biodegradation by sulfate reduction mechanisms, but that substantial sulfate mass may be required to achieve success at full-scale implementation.

## 4. Discussion

Natural or enhanced bioremediation treatment strategies can be favorable approaches for contaminated site management as natural microorganisms present at impacted sites are capable of reducing (degrading or transforming) concentrations of a wide range of pollutants in soil and groundwater. Increasing application of MBTs to directly measure microbiological processes can reduce site uncertainties to better inform remedial decisions and increase the potential for success of bioremediation. Yet, while many publications and guidance documents extol the advantages of employing MBTs in concert with contaminant chemistry and geochemistry evaluations, MBTs application, implementation strategies, and data interpretation are inconsistent. To aid in demonstration of the utility of MBTs to increase knowledge of relevant subsurface microbiological processes, this study focuses on field-scale application of a standardized and systematic framework that pairs MBTs with traditional contaminant and geochemical analyses.

The previously published framework describing a standardized approach to applying MBTs at the field-scale (Key et al., 2022) was successfully applied at two contaminated sites to assess bioremediation capacity and guide remedial decision-making. Framework application at a site with TCE impacts in groundwater supported design, implementation, and monitoring of full-scale injection of an electron donor to promote reductive dechlorination degradation mechanisms within the groundwater plume. Enumeration of 16S rRNA genes via qPCR for obligate reductive dechlorinating bacteria during a field pilot study demonstrated that injection of electron donor (biostimulation) alone would likely not be sufficient for remedy success and that bioaugmentation with a DHC culture could enhance the native dechlorinating microbial population to achieve remedy at this site. At the second site, framework application using MLOE showed that (1) intrinsic bioremediation of PHCs was occurring in the source area and downgradient plume sufficient to meet groundwater remedial objectives, and (2) that addition of a sulfate to increase electron acceptor availability in the source area can increase bioremediation capacity.

Increased application of a standardized approach that employs MBTs in concert with traditional analyses to assess physical, chemical, and biological processes has the potential to reduce site uncertainties, improve bioremediation effectiveness, and provide site managers and stakeholders greater confidence in contaminated site management. Further, it is hoped and anticipated that the associated framework applied and data herein to contextualize current field-scale use of MBTs, identify areas of improvement, and inform future targeted research. As example, potential areas for future research to further improve field-scale use of MBTs at contaminated sites include demonstrating comparative analysis of metabolomics or proteomics versus qPCR data at field-scale to improve or support bioremediation decision-making or conceptual site model development, and development of machine-learning algorithms to assist or automate data interpretation of field-scale MBT, contaminant, and geochemical data to decrease time between data generation and interpretation to support to more informed site decision-making.

## Data availability statement

The original contributions presented in the study are publicly available. This data can be found here: https://doi.org/10.6084/m9.figshare.21905697.v1.

## Author contributions

AM and TK contributed to conception and design of this perspective. SS and YW refined and sharpened conceptual layout and messaging of content. AM and TK wrote sections of the manuscript. All authors contributed to manuscript review and read and approved the submitted manuscript.

## Funding

Funding and in-kind contributions were provided by ExxonMobil Environmental and Property Solutions Company, Golder Associates USA, Inc., and Imperial Oil Limited. The funder had the following involvement in the study: study design, interpretation of data, and the writing of this article.

## Conflict of interest

AM and SS were employed by Golder Associates USA Inc. YW was employed by Imperial Oil Limited. TK was employed by ExxonMobil Environmental and Property Solutions Company.

## Publisher’s note

All claims expressed in this article are solely those of the authors and do not necessarily represent those of their affiliated organizations, or those of the publisher, the editors and the reviewers. Any product that may be evaluated in this article, or claim that may be made by its manufacturer, is not guaranteed or endorsed by the publisher.
