# Supplementary material for: Increasing in situ bioremediation effectiveness through field-scale application of molecular biological tools
Source: Front Microbiol. 2023 Feb 10;13:1005871. doi: 10.3389/fmicb.2022.1005871 (PMC9950576; doi:10.3389/fmicb.2022.1005871)

Figure SI-1. Site 2 concentration trends for monitoring wells (a) MW-A2 (b) MW-A4, (c) MW-A5, and (d) MW-A6.

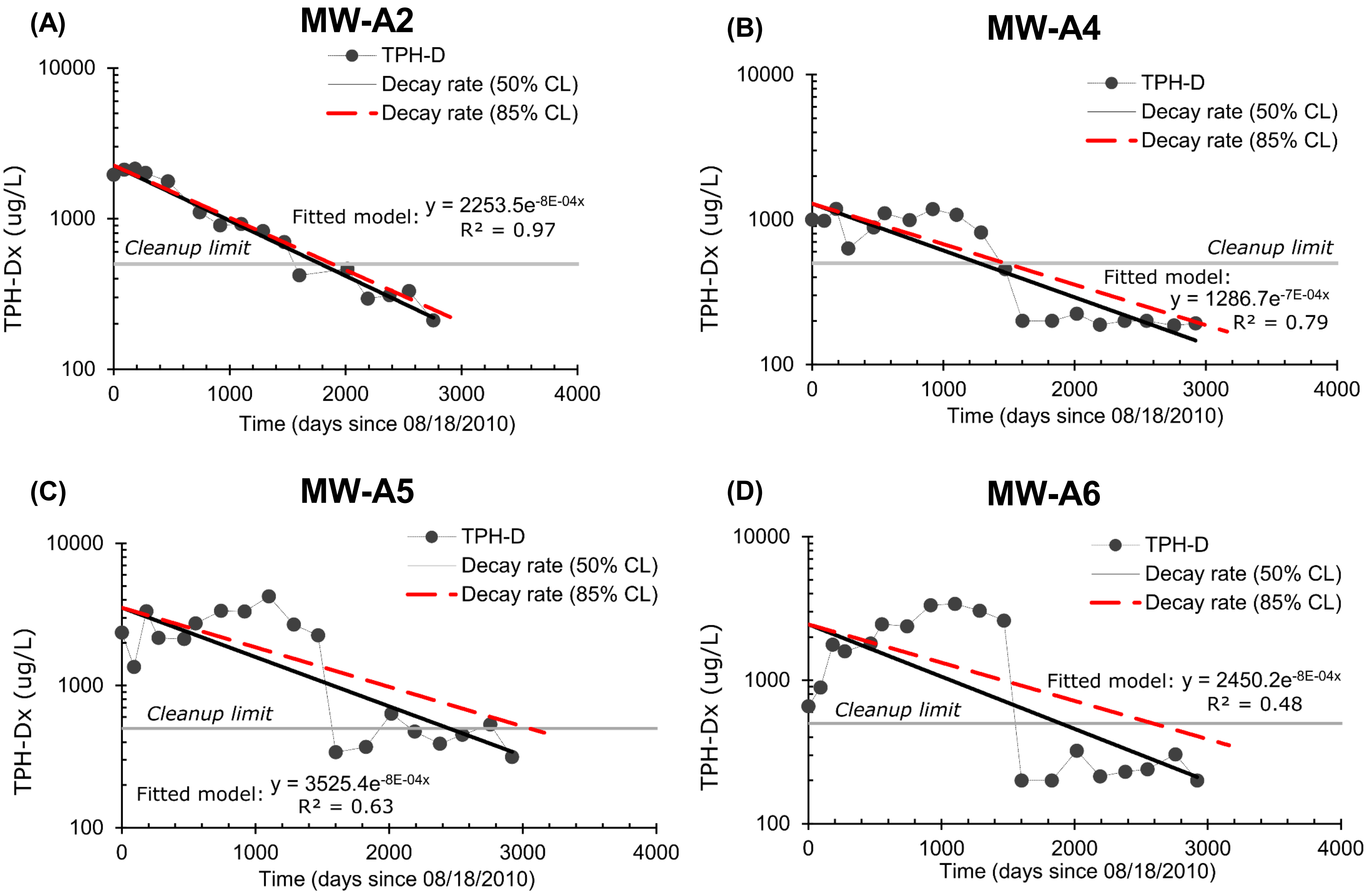

Figure SI-2. Site 2 field pilot study performance monitoring results for the injection wells (IW-1 and IW-2) and the downgradient well (W-15R).

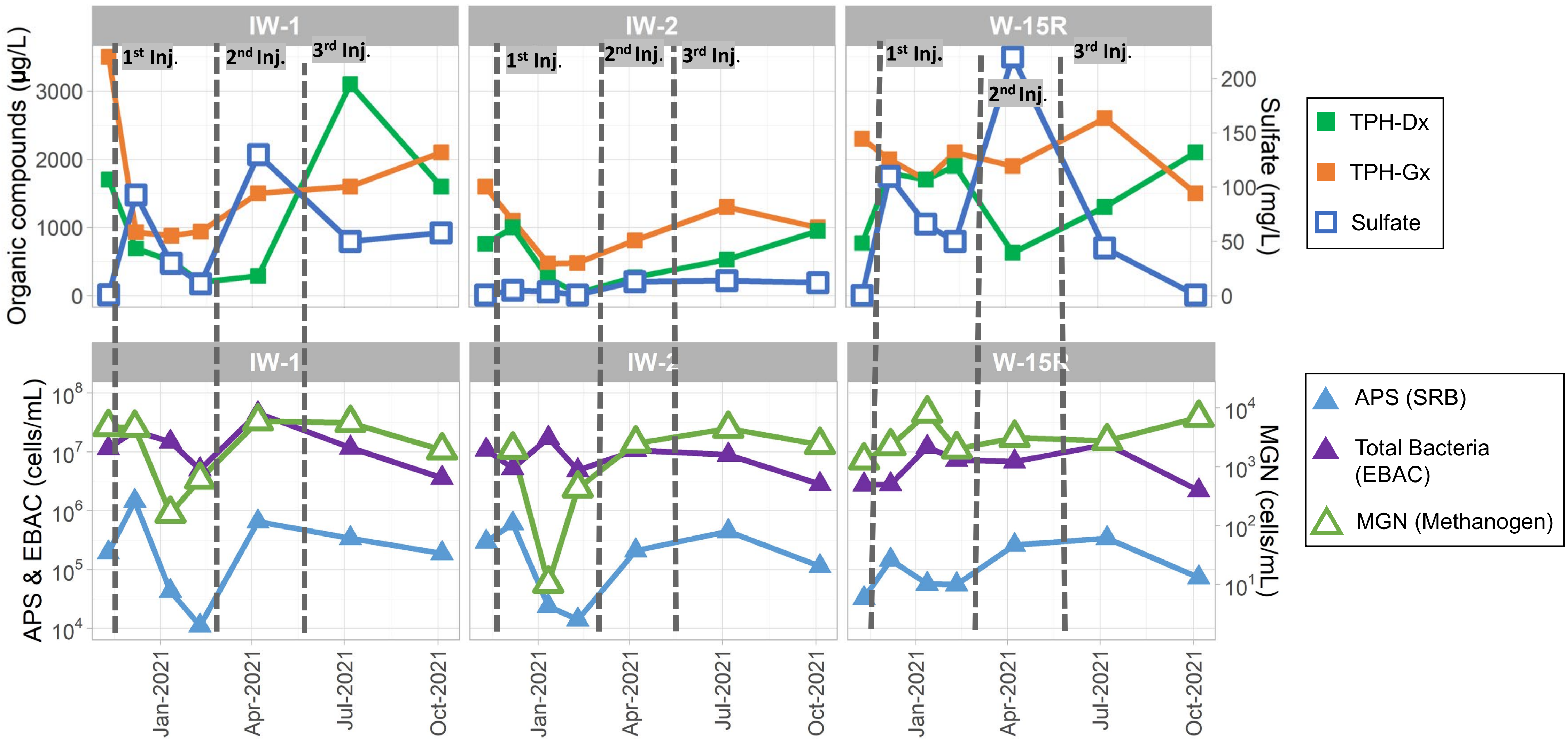

Figure SI-3. Site 2 pilot study performance monitoring trends for upgradient monitoring well OBS-3 and cross-gradient monitoring wells OBS-1 and OBS-2.

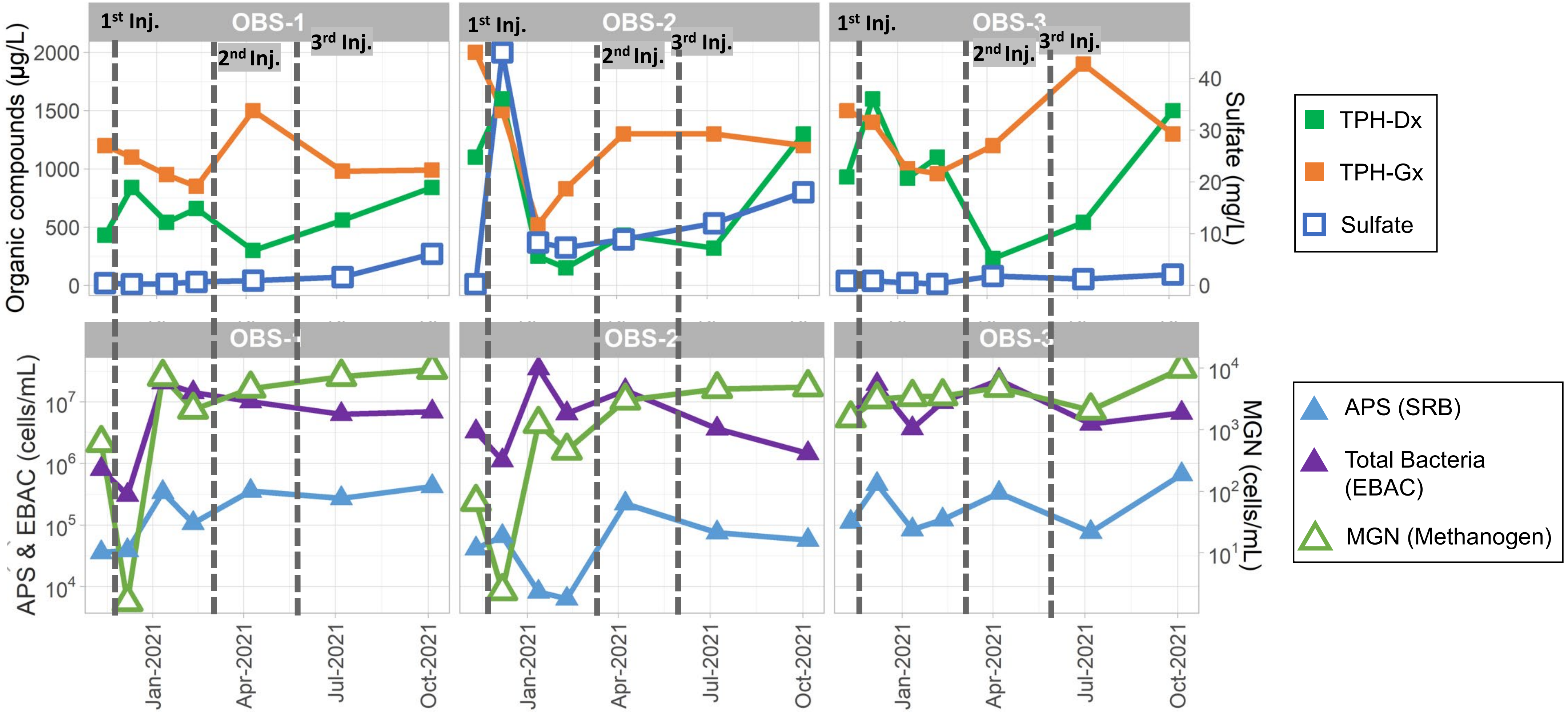

Supplement: Supplementary file 1 [file Image_1.pdf]
