# Supplementary material for: Increasing in situ bioremediation effectiveness through field-scale application of molecular biological tools
Source: Front Microbiol. 2023 Feb 10;13:1005871. doi: 10.3389/fmicb.2022.1005871 (PMC9950576; doi:10.3389/fmicb.2022.1005871)
Supplement: Supplementary file 2 [file Table_1.DOCX]

**Table SI-1.** Electron equivalent estimate for site-specific amendment dosage design.

| **Treatment Area Information** | | | |  |  |  |  |
| --- | --- | --- | --- | --- | --- | --- | --- |
|  | Radius of Influence | 6 | m |  |  |  |  |
|  | Thickness of Treatment Zone | 3 | m |  |  |  |  |
|  | Porosity | 0.3 |  |  |  |  |  |
|  | Bulk Density | 1500 | kg/m^3^ |  |  |  |  |
|  | Total Solids Mass | 356,257 | kg |  |  |  |  |
|  | Total Pore Volume | 102 | m^3^ |  |  |  |  |
|  |  | 101,788 | L |  |  |  |  |
|  | Fraction Organic Carbon | 0.40% | % |  |  |  |  |
|  |  |  |  |  |  |  |  |
| **Biogeochemical Calculations** | | | | | | | |
|  | ***Aqueous Contaminant Electron Equivalents*** | | | | | | |
|  | Chlorinated Contaminant | | Average Concentration (mg/L) | Treatment Area Mass (kg) | Electron Equivalents  (e- equiv/mol) | Total Electron Equivalents (mol) |  |
|  | Tetrachloroethene (PCE) | | 0.1 | 0.01 | 8 | 0.5 |  |
|  | Trichloroethene (TCE) | | 30 | 3.05 | 6 | 110.5 |  |
|  | cis-1,2-Dichloroethene (DCE) | | 5 | 0.51 | 4 | 12.3 |  |
|  | Vinyl Chloride | | 0.5 | 0.05 | 2 | 0.6 |  |
|  | ***Sorbed Contaminant Electron Equivalents*** | | | | | | |
|  | Chlorinated Contaminant | K_oc_ (L/kg) | Estimated Concentration (mg/kg) | Treatment Area Mass (kg) | Electron Equivalents (e- equiv/mol) | Total Electron Equivalents  (mol) |  |
|  | Tetrachloroethene (PCE) | 155 | 0.062 | 0.02 | 8 | 1.1 |  |
|  | Trichloroethene (TCE) | 166 | 19.92 | 7.10 | 6 | 256.8 |  |
|  | cis-1,2-Dichloroethene (DCE) | 35.5 | 0.71 | 0.25 | 4 | 6.1 |  |
|  | Vinyl Chloride | 18.6 | 0.0372 | 0.01 | 2 | 0.2 |  |
|  | ***Background Electron Equivalents*** | | | | | | |
|  | Parameter | | Average Concentration (mg/L) | Treatment Area Mass (kg) | Electron Equivalents (e- equiv/mol) | Total Electron Equivalents (mol) |  |
|  | Oxygen | | 1 | 0.10 | 4 | 12.7 |  |
|  | Nitrate | | 0.5 | 0.05 | 5 | 4.6 |  |
|  | Sulfate | | 20 | 2.04 | 8 | 169.5 |  |
|  |  |  |  |  |  |  |  |
| **Total Electron Equivalents** | | | | | | | |
|  | Aqeuous Contaminant Electron Equivalents = | | | | 124 | e- equiv |  |
|  | Sorbed Contaminant Electron Equivalents = | | | | 264 | e- equiv |  |
|  | Background Electron Equivalents = | | | | 187 | e- equiv |  |
|  | ***Electron Equivalents Subtotal =*** | | | | ***575*** | ***e- equiv*** |  |
|  | Safety Factor = | | | | 4 |  |  |
|  | **Total Electron Equivalents =** | | | | **2,299** | **e- equiv** |  |
